# Supplementary material for: Genetic Background and Antibiotic Resistance Profiles of K. pneumoniae NDM-1 Strains Isolated from UTI, ABU, and the GI Tract, from One Hospital in Poland, in Relation to Strains Nationally and Worldwide
Source: Genes (Basel). 2021 Aug 22;12(8):1285. doi: 10.3390/genes12081285 (PMC8394471; doi:10.3390/genes12081285)
Supplement: Supplementary file 1 [file genes-12-01285-s001.zip › Supplementary materials.pdf]

genes

Supplementary Materials

Title Genetic background and antibiotic resistance profiles of *K. pneumoniae* NDM-1 strains isolated from UTI, ABU and the GI tract, from one hospital in Poland, in relation to strains nationally and worldwide

Magdalena Wysocka [1], Roxana Zamudio [2], Marco R. Oggioni [2], Justyna Gołębiewska [3], Marek Bronk [4] and Beata Krawczyk [1,*]

1 Department of Molecular Biotechnology and Microbiology, Faculty of Chemistry, Gdańsk University of Technology, ul. Narutowicza 11/12, 80-233 Gdańsk, Poland
2 Department of Genetics and Genome Biology, University of Leicester, University Road, Leicester, LE1 7RH, UK
3 Department of Nephrology, Transplantology and Internal Medicine, Medical University of Gdańsk, ul. Dębinki 7, 80-952 Gdańsk, Poland
4 Laboratory of Clinical Microbiology, University Centre for Laboratory Diagnostics, Medical University of Gdańsk Clinical Centre, ul. Dębinki 7, 80-952 Gdańsk, Poland
* Correspondence: beata.krawczyk@pg.edu.pl

Citation: Wysocka, M.; Zamudio, R.; Oggioni, M.R.; Gołębiewska, J.; Bronk, M. Title Genetic background and antibiotic resistance profiles of *K. pneumoniae* NDM-1 strains isolated from UTI, ABU and the GI tract, from one hospital in Poland, in relation to strains nationally and worldwide. *Genes* **2021**, *12*, 1285. https://doi.org/10.3390/genes12081285

Academic Editor(s): Taeok Bae

Received: 19 July 2021
Accepted: 20 August 2021
Published: 22 August 2021

Publisher's Note: MDPI stays neutral with regard to jurisdictional claims in published maps and institutional affiliations.

Copyright: © 2021 by the authors. Submitted for possible open access publication under the terms and conditions of the Creative Commons Attribution (CC BY) license (http://creativecommons.org/licenses/by/4.0/).

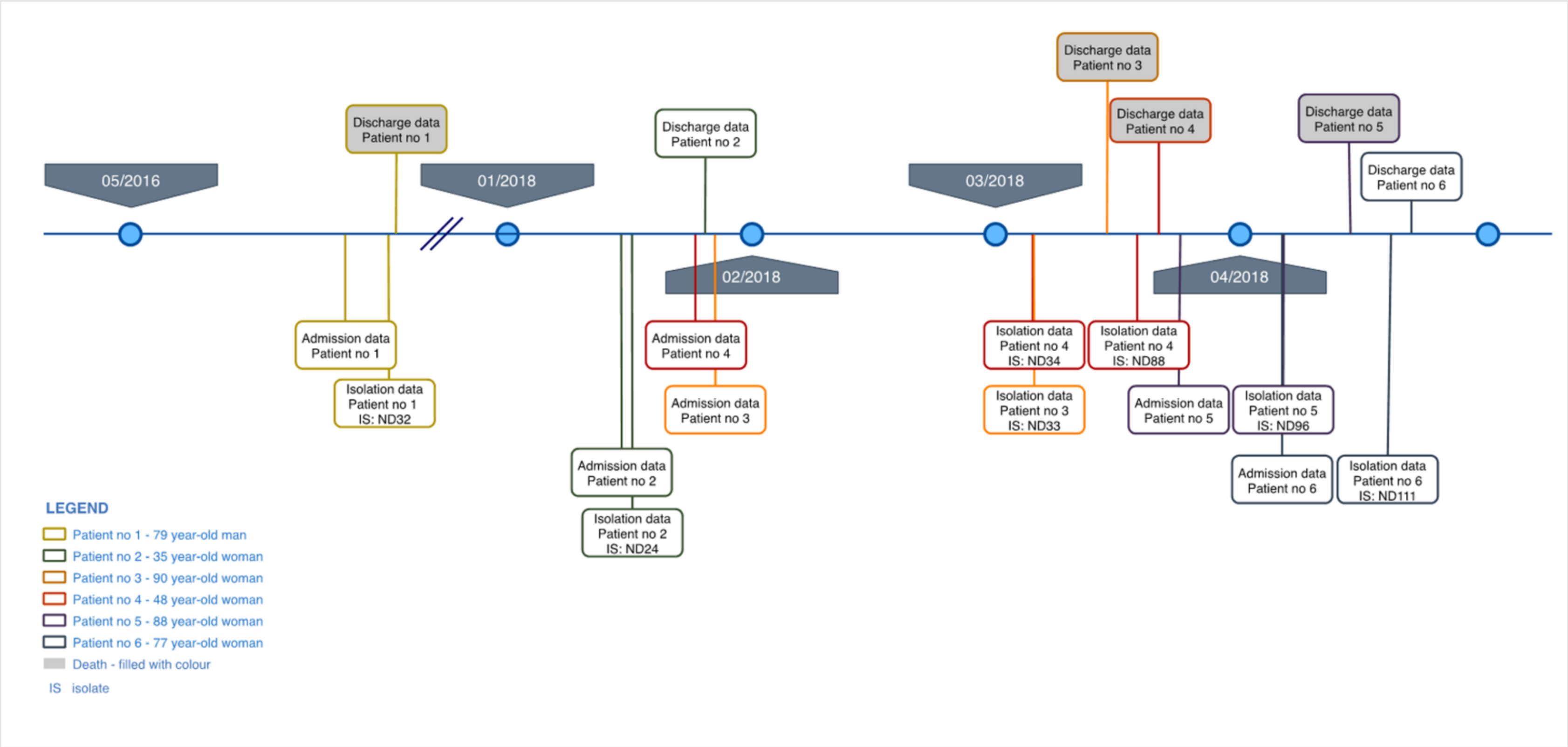

Figure S1. Timeline of events in the epidemiologically linked cases of New Delhi metallo-β-lactamase 1-producing Klebsiella pneumoniae. Dates are given as month/year. Abbreviations: IS, isolate.

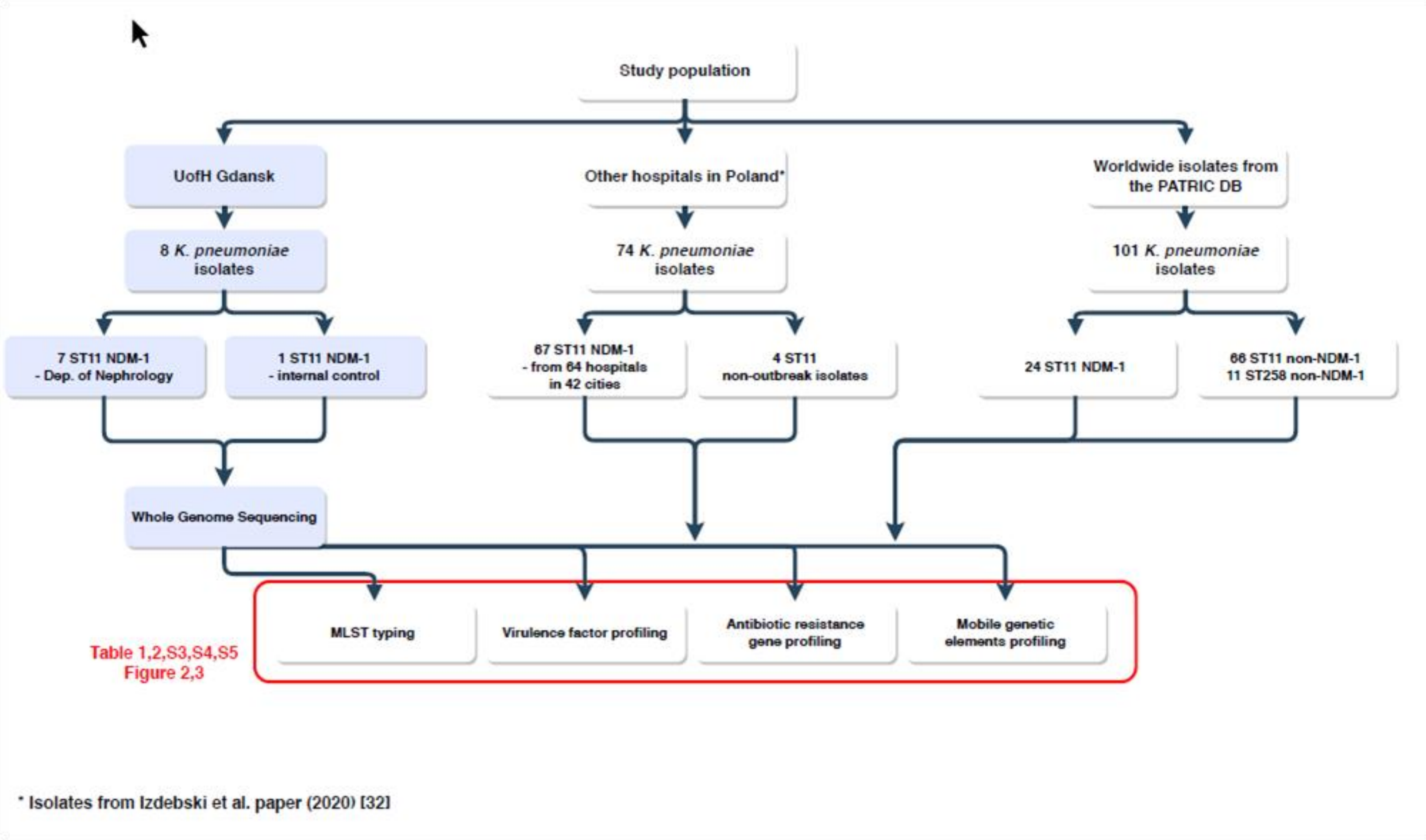

Figure S2. Diagram showing the groups of the analyzed strains in this work.

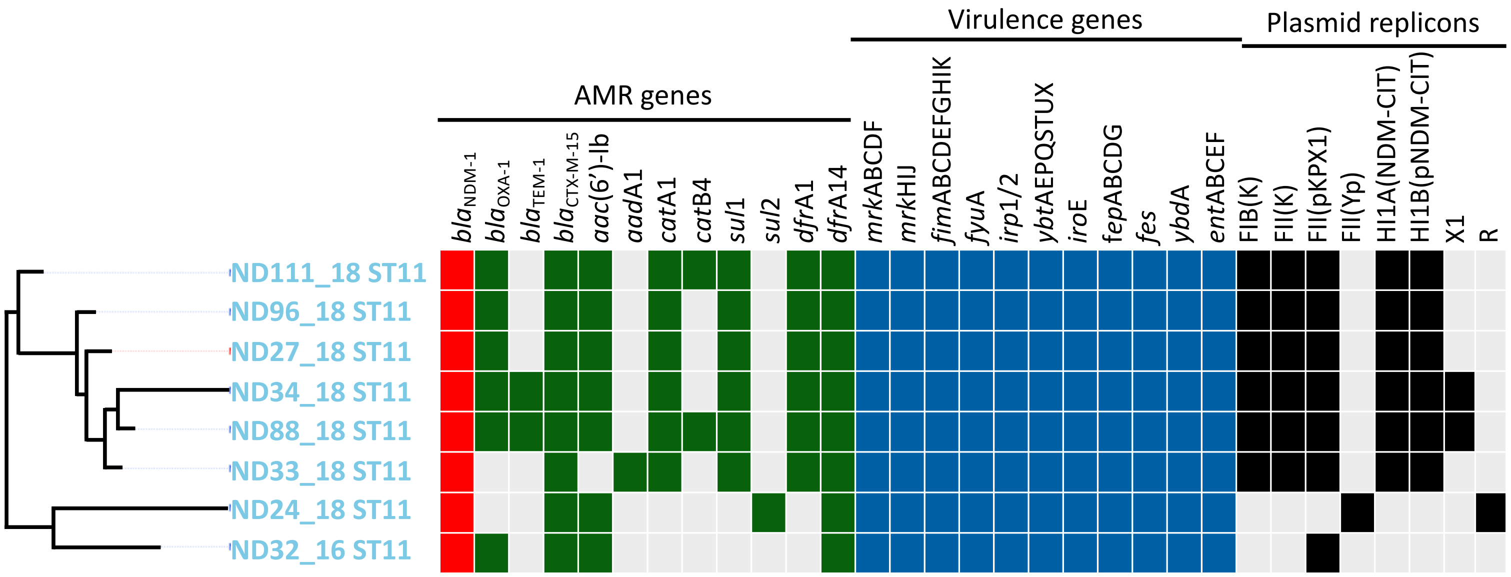

Figure S3. The core-gene phylogenetic tree for eight *K. pneumoniae* isolates from this study. The sequence type (ST) is indicated for each isolate, following the isolate name. In the heatmap the presence of the *bla*NDM-1 gene is indicated by red (present) or gray (absent) and the presence/absence profile of the genotype for genes encoding antimicrobial resistance (green – present, gray – absent), genes encoding virulence determinants (blue – present, gray – absent) and plasmid replicons is indicated.

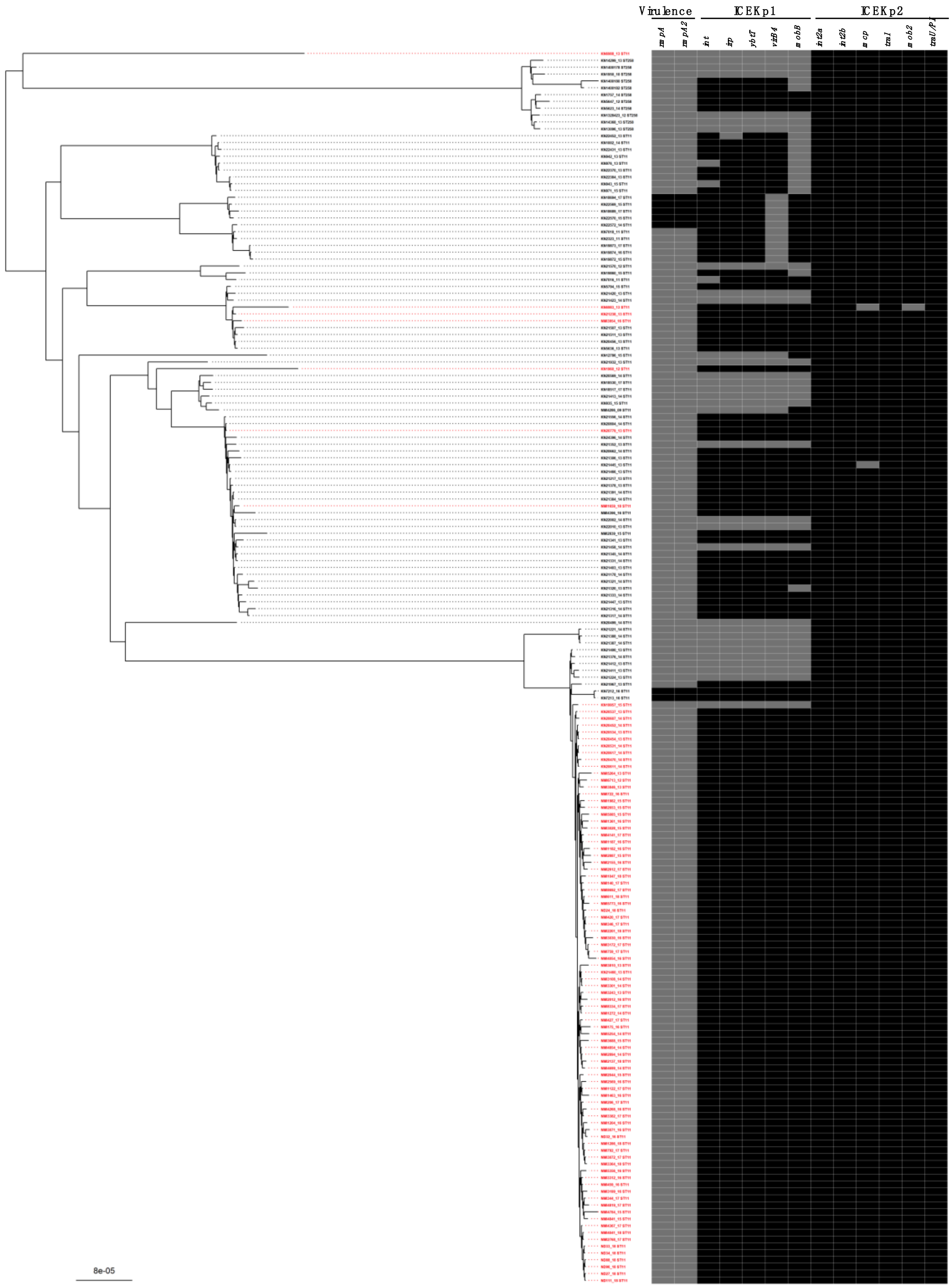

Figure S4. Occurrence of integrative conjugation elements (ICEKp1 and ICEKp2) in clinical isolates of K. pneumoniae. Genome core phylogenetic tree generated for 180 K. pneumoniae isolates. The color of the isolate corresponds to the presence or absence of the blaNDM-1 gene (red – present, black – absent). The sequence type (ST) is indicated for each isolate after the isolate name. The presence and coexistence of ICEKp1 and ICEKp2 (marker genes) is shown in the heatmap on the right (black – present, gray – absent). The virulence related genes rmpA and rmpA2 were also included in the analysis to determine their correlation with the presence / absence of ICEKp1 and ICEKp2. The scale bar shown in the lower left corner shows the average number of substitutions per locus.

Table S1. Strain metadata. Characteristics of the single isolates, including collection details, genome assembly statistics, genomic features.

Assembly/Sample Name	Biosample accession	Country	Hospital	Year	Month	Source	Graft	Infection Status	Age	Sex	ST	# contigs	Largest contig	Total length	GC (%)	N50	N75	L50	L75	# N's per 100 kbp	num_seqs	sum_len	min_len	avg_len	max_len
ND27	SAMN17168596	Poland	Torun	2018	5	urine	0	infection	ND	ND	11	138	483179	5966290	56.84	239522	101300	9	18	0.00	720,384	162,044,155	36	224.9	251
ND96	SAMN17168597	Poland	Gdansk	2018	4	urine	0	infection	ND	K	11	113	804016	5959156	56.81	239630	100790	9	17	0.00	651,043	150,425,106	36	231.1	251
ND88	SAMN17168598	Poland	Gdansk	2018	3	urine	1	colonization	47	K	11	136	483261	6006090	56.72	239522	99546	10	20	0.00	645,02	143,147,694	36	221.9	251
ND32	SAMN17168599	Poland	Gdansk	2016	5	urine	0	infection	ND	M	11	98	483251	5441546	57.37	203937	101300	9	17	0.00	523,802	116,651,684	36	222.7	251
ND111	SAMN17168600	Poland	Gdansk	2018	4	urine	0	colonization	ND	K	11	103	804016	5953882	56.82	241563	101300	8	17	0.00	651,012	142,164,105	36	218.4	251
ND24	SAMN17168601	Poland	Gdansk	2018	1	urine	1	colonization	34	K	11	95	507788	5537205	57.30	258257	142128	8	16	0.00	710,264	168,737,035	36	237.6	251
ND33	SAMN17168602	Poland	Gdansk	2018	3	anal swab	0	colonization	ND	K	11	112	491481	5956422	56.82	248278	153777	9	16	0.00	800,507	187,207,483	36	233.9	251
ND34	SAMN17168603	Poland	Gdansk	2018	3	anal swab	1	colonization	ND	K	11	117	491539	5999106	56.73	258283	129570	9	17	0.00	524,443	121,940,935	36	232.5	251

Table S2. (EXCEL format). Metadata for all additional bacterial isolates included in the study from PATRIC database and Izdebski et al. paper [32].

Table S3. (EXCEL format) The matrix of the core genome SNPs differences determined against the reference genome from the same ST.

Table S4. *K. pneumoniae* isolates included in this study from PATRIC database–basic epidemiological data and resistomes based on sequencing WGS.

							Acquired antimicrobial resistance genes[a]							
**Isolates Name**	**Sources**	**Year of isolation**	**Country**	**Host health**	**ST**	**Plasmid replicon profiles**	**β-lactams**	**aminoglycosides[b]**	**fluoroquinolones[b]**	**macrolide, linosamide, streptogramin antibiotics**	**phenicols**	**sulphonamides**	**tetracycline**	**trimethoprim**
KN2323	urine	2011	China	ND	11	CoIRNAI, FII(pHN7A8), L, R	blaKPC-2	*aadA2*	-	-	*catA2*	-	-	-
KN7016	blood	2011	China	sepsis	11	FIB(K), FII(K), I1-I(Gamma), R	blaCTX-M-55	-	*qnrB4*, *qnrS1*	*mph*(A)	-	*sul1*	-	-
KN7018	urine	2011	China	UTI	11	ColRNAI, FII(pHN7A8), L, N, R	blaKPC-2 blaCTX-M-65, blaTEM-1	*aac(6')-Ib-cr*, *aadA16*, *aadA2*	*aac(6')-Ib-cr*	-	*catA2*	*sul1*	*tet*(A)	*dfrA27*
KN21570	urine	2012	Germany	infection	11	FIB(K), FII, FII(K), L, R	blaCTX-M-15, blaOXA-48	*aac(3)-IVa*, *aph(3'')-Ib*, *aph(4)-Ia*, *aph(6)-Id*, *aadA16*, *aadA2*	*qnrS1*	-	*catA2*	*sul1*, *sul3*	-	*dfrA27*

KN20456	urine	2013	Greece	infection	11	Col440I, FIB(K), FII(K), L, N	blaCTX-M-15, blaOXA-1,-48	*aac(6′)-Ib-cr, aac(3)-IIa, aph(3′)-Ia, aadA2*	*aac(6′)-Ib-cr*	*mph*(A)	-	*sul1*	-	*dfrA12, dfrA14*
KN21217	urine	2013	Slovakia	infection	11	FIA(HI1), FIB(K), FII(K), R	blaCTX-M-15, blaOXA-1,-9, blaTEM-1	*aac(6′)-Ib, aac(3)-IIa, aadA2*	*qnrB4*	-	*catA1*	*sul1*	-	*dfrA12, dfrA14*
KN21224	urine	2013	Czech Republic	infection	11	Col(pHAD28), C, FIB(K), FII(K), R	blaCTX-M-15, blaOXA-1, blaTEM-1	*aac(6′)-Ib-cr, aac(3)-IIa, aph(6)-Id*	*aac(6′)-Ib-cr*	-	-	*sul1*	*tet*(D)	*dfrA22*
KN21306	urine	2013	Estonia	infection	11	FIB(K), FII(K)	blaCTX-M-15, blaOXA-1, blaTEM-1	*aac(6′)-Ib-cr, aac(3)-IIa, aadA2*	*aac(6′)-Ib-cr*	-	-	*sul1*	-	-
KN21320	urine	2013	Poland	infection	11	FIB(K), FII(K), FIA(HI1), R	blaCTX-M-15, blaOXA-1,-9, blaTEM-1	*aac(6′)-Ib-cr, aac(3)-IIa, aadA2*	*aac(6′)-Ib-cr, qnrB4*	-	*catA1*	*sul1*	-	*dfrA12, dfrA14*
KN21341	urine	2013	Poland	infection	11	FIB(K), FII(K), FIA(HI1), R	blaCTX-M-15, blaOXA-1	*aac(6′)-Ib-cr, aac(3)-IIa, aadA2*	*aac(6′)-Ib-cr, qnrB4*	-	*catA1*	*sul1*	-	*dfrA12, dfrA14*
KN21352	urine	2013	France	infection	11	FIB(K), FII(K), R	blaOXA-1	*aac(6′)-Ib-cr, aph(3′)-Ia, aadA2*	*aac(6′)-Ib-cr*	*mph*(A)	*catA1, catB3*	*sul1*	-	*dfrA12*
KN21378	urine	2013	Slovakia	infection	11	FIB(K), FII(K), FIA(HI1), R	blaCTX-M-15, blaOXA-1,-9, blaTEM-1	*aac(6′)-Ib, aac(3)-IIa*	-	-	-	-	-	-

KN21400	urine	2013	Slovakia	colonization	11	FIB(K), FII(K), R	blaCTX-M-15, blaOXA-1, blaTEM-1	*aac(6′)-Ib-cr, aac(3)-IIa, aph(3′′)-Ib, aph(6)-Id, aadA2*	*aac(6′)-Ib-cr*	-	*catA1*	*sul1*	-	*dfrA12*
KN21403	urine	2013	Slovakia	colonization	11	FIB(K), FII(K), FIA(HI1), R	blaCTX-M-15, blaOXA-1,-9, blaTEM-1	*aac(6′)-Ib-cr, aac(3)-IIa, aadA2*	*aac(6′)-Ib-cr, qnrB4*	-	*catA1*	*sul1*	-	*dfrA12, dfrA14*
KN21411	urine	2013	Czech Republic	infection	11	Col440l, FIB(K)	blaCTX-M-15, blaTEM-1	*aac(6′)-Ib-cr, aac(3)-IIa*	*aac(6′)-Ib-cr*	-	-	-	*tet*(D)	-
KN21412	urine	2013	Czech Republic	infection	11	FIB(K), R	blaCTX-M-15, blaOXA-1	*aac(6′)-Ib-cr, aac(3)-IIa, aph(3′′)-Ib, aph(6)-Id, aadA2*	*aac(6′)-Ib-cr*	-	*catA1*	*sul1*	-	*dfrA12*
KN21420	urine	2013	Czech Republic	infection	11	FIB(K), FII(K)	blaOXA-1	*aac(6′)-Ib-cr*	*aac(6′)-Ib-cr, qnrB4*	*mph*(A)	*catA1, catB3*	*sul1*	-	-
KN21445	urine	2013	Estonia	infection	11	FIB(K), FII(K), R	blaOXA-1	*aac(6′)-Ib-cr, aph(3′)-Ia, aadA2*	*aac(6′)-Ib-cr, qnrB4*	*mph*(A)	*catA1, catB3*	*sul1*	-	*dfrA12*

KN21447	urine	2013	Poland	infection	11	FIA(HI1), R	blaCTX-M-15, blaOXA-1,-9, blaTEM-1	*aac(6′)-Ib, aac(3)-IIa, aadA2*	*qnrB4*	-	*catA1*	*sul1*	-	*dfrA12, dfrA14*
KN21466	urine	2013	Estonia	colonization	11	FIB(K), FII(K), M1, R	blaCTX-M-15, blaOXA-1, blaTEM-1	*aac(6′)-Ib-cr, aadA2*	*aac(6′)-Ib-cr*	-	*catA1, catB3*	*sul1*	-	*dfrA12*
KN21507	urine	2013	Malta	colonization	11	Col440l, FIB(K), L, N	blaOXA-1,-48	*aac(6′)-Ib-cr, aac(3)-IIa, aph(3′)-Ia, aadA2*	*aac(6′)-Ib-cr*	*mph*(A)	-	-	-	*dfrA12, dfrA14*
KN21511	urine	2013	Malta	infection	11	Col440l, FIB(K), FII(K), L, N	blaOXA-1,-48	*aac(6′)-Ib-cr, aac(3)-IIa, aph(3′)-Ia*	*aac(6′)-Ib-cr*	-	-	-	-	*dfrA14*
KN21932	urine	2013	Italy	infection	11	Col(BS512), ColRNAI, FIB(K), FII(K), R	blaTEM-1	*aac(3)-IVa, aph(4)-Ia, aadA2*	-	-	-	*sul3*	*tet*(A)	-
KN21967	urine	2013	Spain	infection	11	FIB(K), FII(K), R	blaCTX-M-15, blaOXA-1,-48	*aac(6′)-Ib-cr, aac(3)-IIa, aph(3′)-Ia, aadA2*	*aac(6′)-Ib-cr, qnrB1*	*mph*(A)	*catA1*	*sul1*	-	*dfrA12*
KN22010	urine	2013	Hungary	infection	11	FIA(HI1), FIB(K), FII(K), R	blaCTX-M-15, blaOXA-9, blaTEM-1	*aac(6′)-Ib, aac(3)-IIa, aadA2*	*qnrB4*	-	*catA1*	*sul1*	-	*dfrA12*
KN22370	urine	2013	USA	ND	11	ColRNAI, FIB(K), FII(K)	blaKPC-2,blaCTX-M-15,	*aph(3′)-Ia, aadA2*	-	*mph*(A)	-	*sul1, sul3*	-	*dfrA12*

							blaOXA-9, blaTEM-1							
KN22384	urine	2013	USA	ND	11	ColRNAI, FIB(K), FII(K), R	blaKPC-2, blaCTX-M-15, blaOXA-9, blaTEM-1	*aph(3′)-Ia*, *aadA2*	-	*mph*(A)	-	*sul1*, *sul3*	-	*dfrA12*
KN22431	urine	2013	USA	ND	11	ColRNAI, FIB(K), FIB(pQil), FII(K), R, X3	blaKPC-2, blaCTX-M-15, blaOXA-9, blaTEM-1	*aac(3)-IVa*, *aph(3′)-Ia*, *aph(4)-Ia*, *aadA2*	-	*mph*(A)	*catA1*	*sul1*, *sul3*	-	*dfrA12*
KN22452	urine	2013	USA	ND	11	ColRNAI, FIB(K), M1, R	blaOXA-2	*ant(2′′)-Ia*	-	-	-	*sul1*	-	-
KN5636	stool	2013	Greece	ND	11	CoI440I, C, FIB(K), FII(K), L	blaCTX-M-15, blaOXA-10, blaOXA-1,-48, blaTEM-1	*aac(6′)-Ib-cr*, *aac(3)-IIa*, *ant(2′′)-Ia*, *aph(3′′)-Ib*, *aph(3′)-Ia*, *aph(6)-Id*, *aadA2*	*aac(6′)-Ib-cr*	*mph*(A)	-	*sul2*	*tet*(A), *tet*(G)	*dfrA12*
KN942	urine	2013	USA	ND	11	ColRNAI, FIB(K), FII(K), R, X1	blaKPC-2, blaCTX-M-15, blaOXA-9, blaTEM-1	*aph(3′)-Ia*, *aadA2*	-	*mph*(A)	-	*sul1*, *sul2*, *sul3*	-	*dfrA12*
KN976	urine	2013	USA	ND	11	ColRNAI, FIB(K), FII(K), R, X1	blaKPC-2, blaCTX-M-15, blaOXA-9, blaTEM-1	*aph(3′)-Ia*, *aadA2*	-	*mph*(A)	-	*sul1*, *sul2*, *sul3*	-	*dfrA12*

KN20499	urine	2014	Spain	infection	11	FIB(K), N, R	blaTEM-1	*aac(6′)-Ib-cr, aadA16, aph(3′′)-Ib, aph(6)-Id, aadA2*	*aac(6′)-Ib-cr, qnrB19, qnrB6*	-	-	*sul1, sul3*	-	*dfrA27*
KN20569	urine	2014	Portugal	ND	11	ColRNAI, R, X1	blaCTX-M-1, blaOXA-1	*aac(6′)-Ib-cr, aph(3′)-Ia*	*aac(6′)-Ib-cr, qnrB4*	*mph*(A)	*catB3*	*sul1*	-	-
KN20662	urine	2014	Portugal	ND	11	R	blaOXA-1	*aac(6′)-Ib-cr, aph(3′)-Ia*	*aac(6′)-Ib-cr, qnrB4*	*mph*(A)	*catB3*	*sul1*	*tet*(A)	-
KN20804	urine	2014	Romania	infection	11	R	blaOXA-1	*aac(6′)-Ib-cr, aadA2*	*aac(6′)-Ib-cr, qnrB4*	*mph*(A)	*catB3*	*sul1*	*tet*(A)	-
KN21178	urine	2014	Belgium	ND	11	FIB(K), FII(K), FIA(HI1), L, R	blaCTX-M-15, blaOXA-48,-9, blaTEM-1	*aac(6′)-Ib, aac(3)-IIa, aadA2*	-	-	*catA1*	*sul1*	-	*dfrA12*
KN21221	urine	2014	Slovakia	infection	11	ColRNAI, FIB(K), FII(K), R, C	blaCTX-M-15, blaOXA-1, blaTEM-1	*aac(6′)-Ib-cr, aac(3)-IIa, aph(3′′)-Ib, aph(6)-Id*	*aac(6′)-Ib-cr*	-	*catA1*	*sul2*	*tet*(D)	-
KN21316	discharge from the lower respira	2014	Poland	colonization	11	FIB(K), FII(K), FIA(HI1), R	blaCTX-M-15, blaOXA-1,-9, blaTEM-1	*aac(3)-IIa, aadA2*	-	-	*catA1*	*sul1*	-	*dfrA12, dfrA14*

	tory tract													
KN2 1317	urine	2014	Poland	infection	11	FIB(K), FII(K), FIA(HI1), R	blaCTX-M-15, blaOXA-1,-9, blaTEM-1	*aac(6′)-Ib, aac(3)-IIa, aadA2*	-	-	*catA1*	*sul1*	-	*dfrA12, dfrA14*
KN2 1321	urine	2014	Poland	infection	11	FIB(K), FII(K), FIA(HI1), R	blaCTX-M-15, blaOXA-1	*aac(6′)-Ib-cr, aac(3)-IIa, aadA2*	*aac(6′)-Ib-cr, qnrB4*	-	*catA1*	*sul1*	-	*dfrA12, dfrA14*
KN2 1331	discharge from the lower respiratory tract	2014	Poland	infection	11	FIB(K), FII(K), FIA(HI1), R	blaCTX-M-15, blaOXA-1	*aac(6′)-Ib-cr, aac(3)-IIa, aadA2*	*aac(6′)-Ib-cr*	-	*catA1*	*sul1*	-	*dfrA12, dfrA14*
KN2 1333	urine	2014	Poland	infection	11	FIB(K), FII(K), FIA(HI1), R, M2	blaCTX-M-15, blaOXA-1,-9, blaTEM-1	*aac(6′)-Ib, aac(3)-IId, aadA2*	*qnrB4*	-	*catA1*	*sul1*	-	*dfrA12*
KN2 1345	urine	2014	Poland	infection	11	FIB(K), FII(K), FIA(HI1), R	blaCTX-M-15, blaOXA-1	*aac(6′)-Ib-cr, aac(3)-IIa, aadA2*	*aac(6′)-Ib-cr, qnrB4*	-	*catA1*	*sul1*	-	*dfrA12*
KN2 1376	urine	2014	Slovakia	colonization	11	FIB(K), FII(K), R	blaOXA-1	*aac(6′)-Ib-cr, aadA2*	*aac(6′)-Ib-cr*	-	*catA1*	*sul1*	-	*dfrA12*
KN2 1384	urine	2014	Slovakia	colonization	11	Col440l, FIB(K), FII(K), FIA(HI1), R	blaCTX-M-15, blaOXA-1,-9, blaTEM-1	*aac(6′)-Ib, aac(3)-IIa, aadA2*	*qnrB4*	-	*catA1*	*sul1*	-	*dfrA12, dfrA14*

KN21387	urine	2014	Slovakia	infection	11	FIB(K), FII(K), R	blaCTX-M-15, blaOXA-1, blaTEM-1	*aac(6′)-Ib-cr, aph(3′′)-Ib, aph(6)-Id*	*aac(6′)-Ib-cr*	-	-	*sul2*	*tet*(D)	-
KN21388	urine	2014	Slovakia	infection	11	FIB(K), FII(K), R	blaCTX-M-15, blaOXA-1, blaTEM-1	*aac(6′)-Ib-cr, aac(3)-IIa, aph(3′′)-Ib, aph(6)-Id*	*aac(6′)-Ib-cr*	-	*catA1*	*sul2*	*tet*(D)	-
KN21391	urine	2014	Slovakia	colonization	11	FIB(K), FII(K), FIA(HI1), R	blaCTX-M-15, blaOXA-1,-9, blaTEM-1	*aac(6′)-Ib, aac(3)-IIa, aadA2*	*qnrB4*	-	*catA1*	*sul1*	-	*dfrA12*, *dfrA14*
KN21413	urine	2014	France	colonization	11	FIB(K), FII, M2, R	blaCTX-M-3, blaOXA-1, blaTEM-1	*aac(6′)-Ib-cr, aph(3′′)-Ib, aph(3′)-Ia, aph(6)-Id*	*aac(6′)-Ib-cr, qnrB4*	-	*catB3*	*sul1*	-	-
KN21423	urine	2014	Czech Republic	infection	11	FIB(K), FII(K), R	blaOXA-1	*aac(6′)-Ib-cr, aph(3′)-Ia*	*aac(6′)-Ib-cr, qnrB4*	*mph*(A)	*catA1, catB3*	*sul1*	-	-
KN21458	urine	2014	Poland	infection	11	FIB(K), FII(K)	-	*aadA2*	*qnrB4*	-	*catA1*	*sul1*	-	*dfrA12*
KN21556	discharge from the wound	2014	Germany	colonization	11	FIB(K), FII(K), R	blaOXA-1	*aac(6′)-Ib-cr, aph(3′)-Ia, aadA2*	*aac(6′)-Ib-cr, qnrB4*	*mph*(A)	*catA1, catB3*	*sul1*	-	*dfrA12*

KN22002	urine	2014	Hungary	infection	11	FIA(HI1), FIB(K), FII(K), R	blaCTX-M-15, blaOXA-9, blaTEM-1	*aac(6′)-Ib, aac(3)-IIa, aadA2*	*qnrB4*	-	*catA1*	*sul1*	-	*dfrA12*
KN22572	blood	2014	China	bloodstream infection	11	ColRNAI, FII(pHN7A8), HI1B(pNDM-MAR), I1-I(Gamma), R, repB	blaKPC-2, blaCTX-M-14b,blaTEM-1	*aac(6′)-Ib-cr, aadA16*	*aac(6′)-Ib-cr*	-	*catA2*	*sul1*	-	*dfrA27*
KN24396	urine	2014	USA	ND	11	ColRNAI, pKPC-CAV1193	blaKPC-3, blaTEM-1	*aadA2*	*qnrS1*	-	-	*sul1*	-	-
KN1002	urine	2014	USA	ND	11	ColRNAI, FIB(K), FIB(pQil), R	blaKPC-2, blaCTX-M-15, blaOXA-9, blaTEM-1	*aph(3′)-Ia, aadA2*	-	*mph*(A)	-	*sul1, sul3*	-	*dfrA12*
KN12786	urine	2015	Nigeria	ND	11	ColKP3, FIB(K), FIB(pNDM-Mar), FII(K), R, X3	blaCTX-M-15, blaOXA-1,-181, blaTEM-1	*aac(6′)-Ib-cr, aac(3)-IIa, aph(3′′)-Ib, aph(6)-Id*	*aac(6′)-Ib-cr, qnrB2, qnrS1*	*mph*(A)	*catA1*	*sul1*	*tet*(A)	*dfrA15*
KN19860	blood	2015	Brazil	infection	11	ColRNAI, FIB(K), FII(K), N	blaKPC-2, blaCTX-M-2, blaOXA-2, blaTEM-1	*aac(3)-IIa, aadA2*	-	-	*catA1*	*sul1*	-	*dfrA12*
KN19872	stool	2015	China	ND	11	FIB(K), FIB(pKPHS1), FII(pHN7A8), R	blaKPC-2, blaCTX-M-14 blaTEM-1	-	*qnrS1*	-	-	*sul1*	*tet*(A)	*dfrA1*
KN22569	blood	2015	China	bloodstream	11	ColRNAI, FII(pHN7A8),	blaKPC-2, blaCTX-M-147	*aadA2*	-	-	-	-	-	-

				infection		HI1B(pNDM-MAR), R, repB								
KN22570	blood	2015	China	bloodstream infection	11	ColRNAI, FII(pHN7A8), HI1B(pNDM-MAR), repB	blaKPC-2, blaCTX-M-65, blaTEM-1	*aadA2*	-	-	-	-	-	-
KN5794	urine	2015	Thailand	ND	11	Col8282, FIB(K), FII(K), I1-I(Gamma), R	blaCTX-M-15, blaOXA-1, blaTEM-1	*aac(6′)-Ib-cr, aac(3)-IId, aph(3′)-Ia, aadA17*	*aac(6′)-Ib-cr, qnrB4*	*mph*(A)	*catA1, catB3*	*sul1*	-	*dfrA12*
KN935	discharge from the wound	2015	USA	ND	11	FIA(HI1), FIB(K), FII(K), M1, R	blaCTX-M-15, blaOXA-1	*aac(6′)-Ib-cr, aac(3)-IIa, ant(2′′)-Ia, aadA2*	*aac(6′)-Ib-cr, qnrS1*	-	-	*sul1*	*tet*(A)	*dfrA12, dfrA14*
KN943	urine	2015	USA	ND	11	ColRNAI, FIB(K), FII(K), R	blaKPC-2, blaCTX-M-15, blaOXA-9, blaTEM-1	*aph(3′)-Ia, aadA2*	-	*mph*(A)	-	*sul1, sul3*	-	*dfrA12*
KN971	discharge from the wound	2015	USA	ND	11	ColRNAI, FIB(K), FII(K), R	blaKPC-2, blaCTX-M-15, blaOXA-9, blaTEM-1	*aph(3′)-Ia, aadA2*	-	*mph*(A)	-	*sul1, sul3*	-	*dfrA12*

KN19874	stool	2016	Chiny	ND	11	FIB(K), FIB(pKPHS1), FII(pHN7A8), R	blaKPC-2, blaCTX-M-65 blaTEM-1	-	*qnrS1*	-	-	*sul1*	*tet*(A)	*dfrA1*
KN7212	urine	2016	India	bloodstream infection	11	ColKP3, FIB(pQil), FII(K), HI1B(pNDM-MAR), R, repB	blaCTX-M-15, blaOXA-232, blaTEM-1	*aph(3'')-Ib*, *aph(6)-Id*	*qnrB1*	-	-	*sul2*	-	-
KN7213	blood	2016	India	bloodstream infection	11	ColKP3, FIB(pQil), FII(K), HI1B(pNDM-MAR), R, repB	blaCTX-M-15, blaOXA-232, blaTEM-1	*aph(3'')-Ib*, *aph(6)-Id*	*qnrB1*	-	-	*sul2*	-	-
KN18517	urine	2017	USA	infection	11	C	-	-	-	-	-	*sul2*	-	-
KN18530	blood	2017	USA	infection	11	C, R	blaOXA-10	*aadA2*	-	-	-	*sul1*, *sul2*	*tet*(D)	*dfrA14*
KN18689	stool	2017	China	ND	11	ColRNAI, FII(pHN7A8),HI1B(pNDM-MAR), R, repB	blaKPC-2, blaCTX-M-65, blaTEM-1	*aadA2*	*qnrS1*	-	*catA2*	*sul2*	*tet*(A)	*dfrA14*
KN18694	stool	2017	China	ND	11	ColRNAI, FII(pHN7A8), N, R, repB	blaKPC-2, blaCTX-M-3, blaTEM-1	*aadA2*, *armA*	-	*mph*(E), *msr*(E)	*catB8*		-	*dfrA12*
KN19873	stool	2017	China	ND	11	FIB(K), FIB(pKPHS1), FII(pHN7A8), R	blaKPC-2, blaCTX-M-90 blaTEM-1	-	*qnrS1*	-	-	*sul1*	*tet*(A)	*dfrA1*
KN1950	urine	2010	Norway	ND	258	ColRNAI, FIB(K), FII(K), FII(Yp), I2(Delta)	blaKPC-3, blaOXA-9	*aph(3')-Ia*, *aadA2*	-	*mph*(A)	*catA1*	*sul1*	-	*dfrA12*

KN1328423	urine	2012	USA	ND	258	ColRNAI, FIB(K), FII(K), R, X3	blaKPC-2,	*aac(6′)-Ib, aac(3)-IVa, aph(4)-Ia, aadA2*	-	*mph*(A)	*catA1*	*sul1, sul3*	-	*dfrA12*
KN5647	urine	2012	Greece	ND	258	ColRNAI, FIB(K), FIB(pQil), FII(K), C, X3	blaKPC-2, blaOXA-10,-9	*aac(6′)-Ib, ant(2′′)-Ia, aph(3′′)-Ib, aph(3′)-Ia, aph(6)-Id, aadA2*	-	*mph*(A)	*catA1*	*sul2*	*tet*(A), *tet*(G)	*dfrA12, dfrA14, dfrA23*
KN13096	urine	2013	USA	ND	258	ColRNAI, FIB(K), FIB(pQil), FII(K)	blaKPC-2, blaOXA-9	*aac(6′)-Ib, aph(3′)-Ia, aadA2*	-	*mph*(A)	*catA1*	*sul1*	-	*dfrA12*
KN14299	urine	2013	USA	ND	258	ColRNAI, X3	blaTEM-1	*aac(6′)-Ib*	-	-	-	-	-	-
KN14368	urine	2013	USA	ND	258	ColRNAI, FIB(K), FIB(pQil), FII(K), X3	blaKPC-2, blaOXA-9	*aac(6′)-Ib, aph(3′)-Ia*	-	*mph*(A)	*catA1*	*sul1*	-	*dfrA12*
KN1757	urine	2014	Australia	UTI	258	ColRNAI, FIB(K), FII(K), FIB(pQil), X3	blaKPC-2, blaOXA-9	*aac(6′)-Ib, aph(3′)-Ia, aadA2*	-	*mph*(A)	*catA1*	*sul1*	-	*dfrA12*
KN5623	stool	2014	Greece	ND	258	ColRNAI, FIB(K), FII(K), M1, X3	blaKPC-2,	*aac(6′)-Ib, aph(3′)-Ia, aadA2*	-	*mph*(A)	-	*sul1*	-	*dfrA12*

KN1 400178	urine	ND	USA	ND	258	ColRNAI, FIB(K), FII(K), FII(Yp), X3	blaKPC-3, blaOXA-9, blaTEM-1	*aph(3′′)-Ib*, *aph(6)-Id*, *aadA2*	-	*mph*(A)	*catA1*	*sul1*, *sul2*	-	*dfrA12*, *dfrA14*
KN1 400180	urine	ND	USA	ND	258	ColRNAI, FIA(pBK30683), FIB(K), X3	blaKPC-3, blaOXA-9	*aph(3′′)-Ib*, *aph(6)-Id*	-	*mph*(A)	-	*sul2*	-	*dfrA14*
KN1 400182	urine	ND	USA	ND	258	FIA(pBK30683), FIB(K), X3	blaKPC-3,	*aph(3′)-Ia*	-	*mph*(A)	-	-	-	*dfrA14*

[a] Only acquired resistance genes are shown, as identified by ResFinder 3.1 [34]. [b] The *aac(6′)-Ib-cr* gene shown in the 'aminoglycosides' and 'fluoroquinolones' columns is the same gene, conferring resistance to both classes of antimicrobials.

Table S5. Occurrence of integrative conjugation elements (ICEKp1 and ICEKp2) in clinical isolates of *K. pneumoniae* sequenced in this study and from PATRIC database and from Izdebski et al. paper [32]. (0 – absence, 1 - presence).

Sample Name	*rmpA*	*rmpA2*	*int*	*irp*	*ybtT*	*virB4*	*mobB*	*int2a*	*int2b*	*mcp*	*tral*	*mob2*	*traU/PI*
**ND32 ST11**	0	0	1	1	1	1	1	1	1	1	1	1	1
**ND111 ST11**	0	0	1	1	1	1	1	1	1	1	1	1	1
**ND27 ST11**	0	0	1	1	1	1	1	1	1	1	1	1	1
**ND96 ST11**	0	0	1	1	1	1	1	1	1	1	1	1	1
**ND88 ST11**	0	0	1	1	1	1	1	1	1	1	1	1	1
**ND24 ST11**	0	0	1	1	1	1	1	1	1	1	1	1	1
**ND33 ST11**	0	0	1	1	1	1	1	1	1	1	1	1	1
**ND34 ST11**	0	0	1	1	1	1	1	1	1	1	1	1	1
KN1002 ST11	0	0	1	1	1	1	0	1	1	1	1	1	1
KN12786 ST11	0	0	0	0	0	0	1	1	1	1	1	1	1
KN13096 ST258	0	0	0	0	0	0	0	1	1	1	1	1	1
KN1328423 ST258	0	0	0	0	0	0	0	1	1	1	1	1	1
KN1400178 ST258	0	0	0	0	0	0	0	1	1	1	1	1	1
KN1400180 ST258	0	0	1	1	1	1	0	1	1	1	1	1	1
KN1400182 ST258	0	0	1	1	1	1	0	1	1	1	1	1	1
KN14299 ST258	0	0	0	0	0	0	0	1	1	1	1	1	1
KN14368 ST258	0	0	0	0	0	0	0	1	1	1	1	1	1
KN1757 ST258	0	0	1	1	1	1	1	1	1	1	1	1	1
KN18517 ST11	0	0	0	0	0	0	0	1	1	1	1	1	1
KN18530 ST11	0	0	0	0	0	0	0	1	1	1	1	1	1
KN18689 ST11	1	1	1	1	1	0	1	1	1	1	1	1	1
KN18694 ST11	1	1	1	1	1	0	1	1	1	1	1	1	1
KN1950 ST258	0	0	0	0	0	0	0	1	1	1	1	1	1
KN1960 ST11	0	0	1	1	1	1	1	1	1	1	1	1	1
KN19860 ST11	0	0	1	1	1	1	0	1	1	1	1	1	1
KN19872 ST11	0	0	1	1	1	0	1	1	1	1	1	1	1
KN19873 ST11	0	0	1	1	1	0	1	1	1	1	1	1	1
KN19874 ST11	0	0	1	1	1	0	1	1	1	1	1	1	1
KN19957 ST11	0	0	0	0	0	0	0	1	1	1	1	1	1
KN20452 ST11	0	0	1	1	1	1	1	1	1	1	1	1	1

KN20454 ST11	0	0	1	1	1	1	1	1	1	1	1	1	1
KN20456 ST11	0	0	1	1	1	1	1	1	1	1	1	1	1
KN20470 ST11	0	0	1	1	1	1	1	1	1	1	1	1	1
KN20499 ST11	0	0	0	0	0	0	0	1	1	1	1	1	1
KN20531 ST11	0	0	1	1	1	1	1	1	1	1	1	1	1
KN20534 ST11	0	0	1	1	1	1	1	1	1	1	1	1	1
KN20537 ST11	0	0	1	1	1	1	1	1	1	1	1	1	1
KN20569 ST11	0	0	0	0	0	0	0	1	1	1	1	1	1
KN20607 ST11	0	0	1	1	1	1	1	1	1	1	1	1	1
KN20611 ST11	0	0	1	1	1	1	1	1	1	1	1	1	1
KN20617 ST11	0	0	1	1	1	1	1	1	1	1	1	1	1
KN20662 ST11	0	0	1	1	1	1	1	1	1	1	1	1	1
KN20779 ST11	0	0	1	1	1	1	1	1	1	1	1	1	1
KN20804 ST11	0	0	1	1	1	1	1	1	1	1	1	1	1
KN21178 ST11	0	0	1	1	1	1	1	1	1	1	1	1	1
KN21217 ST11	0	0	1	1	1	1	1	1	1	1	1	1	1
KN21221 ST11	0	0	0	0	0	0	0	1	1	1	1	1	1
KN21224 ST11	0	0	0	0	0	0	0	1	1	1	1	1	1
KN21238 ST11	0	0	1	1	1	1	1	1	1	1	1	1	1
KN21306 ST11	0	0	1	1	1	1	1	1	1	1	1	1	1
KN21316 ST11	0	0	1	1	1	1	1	1	1	1	1	1	1
KN21317 ST11	0	0	1	1	1	1	1	1	1	1	1	1	1
KN21320 ST11	0	0	1	1	1	1	0	1	1	1	1	1	1
KN21321 ST11	0	0	1	1	1	1	1	1	1	1	1	1	1
KN21331 ST11	0	0	1	1	1	1	1	1	1	1	1	1	1
KN21333 ST11	0	0	1	1	1	1	1	1	1	1	1	1	1
KN21341 ST11	0	0	1	1	1	1	1	1	1	1	1	1	1
KN21345 ST11	0	0	1	1	1	1	1	1	1	1	1	1	1
KN21352 ST11	0	0	0	0	0	0	0	1	1	1	1	1	1
KN21376 ST11	0	0	0	0	0	0	0	1	1	1	1	1	1
KN21378 ST11	0	0	1	1	1	1	1	1	1	1	1	1	1
KN21384 ST11	0	0	1	1	1	1	1	1	1	1	1	1	1
KN21387 ST11	0	0	0	0	0	0	0	1	1	1	1	1	1
KN21388 ST11	0	0	0	0	0	0	0	1	1	1	1	1	1
KN21391 ST11	0	0	1	1	1	1	1	1	1	1	1	1	1
KN21400 ST11	0	0	0	0	0	0	0	1	1	1	1	1	1
KN21403 ST11	0	0	1	1	1	1	1	1	1	1	1	1	1
KN21411 ST11	0	0	0	0	0	0	0	1	1	1	1	1	1
KN21412 ST11	0	0	0	0	0	0	0	1	1	1	1	1	1
KN21413 ST11	0	0	0	0	0	0	0	1	1	1	1	1	1
KN21420 ST11	0	0	0	0	0	0	0	1	1	1	1	1	1
KN21423 ST11	0	0	0	0	0	0	0	1	1	1	1	1	1

KN21445 ST11	0	0	1	1	1	1	1	1	1	0	1	1	1
KN21447 ST11	0	0	1	1	1	1	1	1	1	1	1	1	1
KN21458 ST11	0	0	0	0	0	0	0	1	1	1	1	1	1
KN21460 ST11	0	0	1	1	1	1	1	1	1	1	1	1	1
KN21466 ST11	0	0	1	1	1	1	1	1	1	1	1	1	1
KN21507 ST11	0	0	1	1	1	1	1	1	1	1	1	1	1
KN21511 ST11	0	0	1	1	1	1	1	1	1	1	1	1	1
KN21556 ST11	0	0	1	1	1	1	1	1	1	1	1	1	1
KN21570 ST11	0	0	0	0	0	0	0	1	1	1	1	1	1
KN21932 ST11	0	0	0	0	0	0	0	1	1	1	1	1	1
KN21967 ST11	0	0	1	1	1	1	1	1	1	1	1	1	1
KN22002 ST11	0	0	0	0	0	0	0	1	1	1	1	1	1
KN22010 ST11	0	0	0	0	0	0	0	1	1	1	1	1	1
KN22370 ST11	0	0	1	1	1	1	0	1	1	1	1	1	1
KN22384 ST11	0	0	1	1	1	1	0	1	1	1	1	1	1
KN22431 ST11	0	0	1	1	1	1	0	1	1	1	1	1	1
KN22452 ST11	0	0	1	0	1	1	0	1	1	1	1	1	1
KN22569 ST11	1	1	1	1	1	0	1	1	1	1	1	1	1
KN22570 ST11	1	1	1	1	1	0	1	1	1	1	1	1	1
KN22572 ST11	1	1	1	1	1	0	1	1	1	1	1	1	1
KN2323 ST11	0	0	1	1	1	0	1	1	1	1	1	1	1
KN24396 ST11	0	0	1	1	1	1	1	1	1	1	1	1	1
KN5623 ST258	0	0	1	1	1	1	1	1	1	1	1	1	1
KN5636 ST11	0	0	1	1	1	1	1	1	1	1	1	1	1
KN5647 ST258	0	0	1	1	1	1	1	1	1	1	1	1	1
KN5794 ST11	0	0	1	1	1	1	1	1	1	1	1	1	1
KN6983 ST11	0	0	1	1	1	1	1	1	1	0	1	0	1
KN6988 ST11	0	0	0	0	0	0	0	1	1	1	1	1	1
KN7016 ST11	0	0	0	1	1	1	1	1	1	1	1	1	1
KN7018 ST11	0	0	1	1	1	0	1	1	1	1	1	1	1
KN7212 ST11	1	1	1	1	1	1	1	1	1	1	1	1	1
KN7213 ST11	1	1	1	1	1	1	1	1	1	1	1	1	1
KN935 ST11	0	0	0	0	0	0	0	1	1	1	1	1	1
KN942 ST11	0	0	1	1	1	1	0	1	1	1	1	1	1
KN943 ST11	0	0	0	1	1	1	0	1	1	1	1	1	1
KN971 ST11	0	0	1	1	1	1	0	1	1	1	1	1	1
KN976 ST11	0	0	0	1	1	1	0	1	1	1	1	1	1
NMI6713_12	0	0	1	1	1	1	1	1	1	1	1	1	1
NMI3243_13	0	0	1	1	1	1	1	1	1	1	1	1	1
NMI3810_13	0	0	1	1	1	1	1	1	1	1	1	1	1
NMI3846_13	0	0	1	1	1	1	1	1	1	1	1	1	1
NMI5264_13	0	0	1	1	1	1	1	1	1	1	1	1	1

NMI1272_14	0	0	1	1	1	1	1	1	1	1	1	1	1
NMI2894_14	0	0	1	1	1	1	1	1	1	1	1	1	1
NMI3108_14	0	0	1	1	1	1	1	1	1	1	1	1	1
NMI3301_14	0	0	1	1	1	1	1	1	1	1	1	1	1
NMI4699_14	0	0	1	1	1	1	1	1	1	1	1	1	1
NMI4854_14	0	0	1	1	1	1	1	1	1	1	1	1	1
NMI5254_14	0	0	1	1	1	1	1	1	1	1	1	1	1
NMI1982_15	0	0	1	1	1	1	1	1	1	1	1	1	1
NMI2044_15	0	0	1	1	1	1	1	1	1	1	1	1	1
NMI2653_15	0	0	1	1	1	1	1	1	1	1	1	1	1
NMI2887_15	0	0	1	1	1	1	1	1	1	1	1	1	1
NMI3028_15	0	0	1	1	1	1	1	1	1	1	1	1	1
NMI3688_15	0	0	1	1	1	1	1	1	1	1	1	1	1
NMI4784_15	0	0	1	1	1	1	1	1	1	1	1	1	1
NMI4841_15	0	0	1	1	1	1	1	1	1	1	1	1	1
NMI5085_15	0	0	1	1	1	1	1	1	1	1	1	1	1
NMI175_16	0	0	1	1	1	1	1	1	1	1	1	1	1
NMI459_16	0	0	1	1	1	1	1	1	1	1	1	1	1
NMI722_16	0	0	1	1	1	1	1	1	1	1	1	1	1
NMI1182_16	0	0	1	1	1	1	1	1	1	1	1	1	1
NMI1187_16	0	0	1	1	1	1	1	1	1	1	1	1	1
NMI1204_16	0	0	1	1	1	1	1	1	1	1	1	1	1
NMI1361_16	0	0	1	1	1	1	1	1	1	1	1	1	1
NMI1463_16	0	0	1	1	1	1	1	1	1	1	1	1	1
NMI2012_16	0	0	1	1	1	1	1	1	1	1	1	1	1
NMI2155_16	0	0	1	1	1	1	1	1	1	1	1	1	1
NMI2569_16	0	0	1	1	1	1	1	1	1	1	1	1	1
NMI3030_16	0	0	1	1	1	1	1	1	1	1	1	1	1
NMI3071_16	0	0	1	1	1	1	1	1	1	1	1	1	1
NMI3199_16	0	0	1	1	1	1	1	1	1	1	1	1	1
NMI3312_16	0	0	1	1	1	1	1	1	1	1	1	1	1
NMI4054_16	0	0	1	1	1	1	1	1	1	1	1	1	1
NMI4268_16	0	0	1	1	1	1	1	1	1	1	1	1	1
NMI5356_16	0	0	1	1	1	1	1	1	1	1	1	1	1
NMI5773_16	0	0	1	1	1	1	1	1	1	1	1	1	1
NMI140_17	0	0	1	1	1	1	1	1	1	1	1	1	1
NMI206_17	0	0	1	1	1	1	1	1	1	1	1	1	1
NMI344_17	0	0	1	1	1	1	1	1	1	1	1	1	1
NMI346_17	0	0	1	1	1	1	1	1	1	1	1	1	1
NMI420_17	0	0	1	1	1	1	1	1	1	1	1	1	1
NMI427_17	0	0	1	1	1	1	1	1	1	1	1	1	1
NMI759_17	0	0	1	1	1	1	1	1	1	1	1	1	1

NMI792_17	0	0	1	1	1	1	1	1	1	1	1	1	1
NMI1122_17	0	0	1	1	1	1	1	1	1	1	1	1	1
NMI2612_17	0	0	1	1	1	1	1	1	1	1	1	1	1
NMI2768_17	0	0	1	1	1	1	1	1	1	1	1	1	1
NMI3072_17	0	0	1	1	1	1	1	1	1	1	1	1	1
NMI3172_17	0	0	1	1	1	1	1	1	1	1	1	1	1
NMI3382_17	0	0	1	1	1	1	1	1	1	1	1	1	1
NMI4141_17	0	0	1	1	1	1	1	1	1	1	1	1	1
NMI4367_17	0	0	1	1	1	1	1	1	1	1	1	1	1
NMI4819_17	0	0	1	1	1	1	1	1	1	1	1	1	1
NMI8334_17	0	0	1	1	1	1	1	1	1	1	1	1	1
NMI8692_17	0	0	1	1	1	1	1	1	1	1	1	1	1
NMI611_18	0	0	1	1	1	1	1	1	1	1	1	1	1
NMI1047_18	0	0	1	1	1	1	1	1	1	1	1	1	1
NMI1286_18	0	0	1	1	1	1	1	1	1	1	1	1	1
NMI1659_18	0	0	1	1	1	1	1	1	1	1	1	1	1
NMI2137_18	0	0	1	1	1	1	1	1	1	1	1	1	1
NMI2201_18	0	0	1	1	1	1	1	1	1	1	1	1	1
NMI3364_18	0	0	1	1	1	1	1	1	1	1	1	1	1
NMI4041_18	0	0	1	1	1	1	1	1	1	1	1	1	1
NMI3954_16	0	0	1	1	1	1	1	1	1	1	1	1	1
NMI2839_15	0	0	1	1	1	1	1	1	1	1	1	1	1
NMI4286_09	0	0	0	0	0	0	1	1	1	1	1	1	1
NMI4396_16	0	0	1	1	1	1	1	1	1	1	1	1	1

Table S6. *K. pneumoniae* isolates (non NDM-1) from PATRIC database included in the study – serotypes data and virulomes (0 – absence, 1 - presence)

Sample Name	ST	Serotypes		Virulence factors										
		Capsule (K) antigen types	LPS (O) antigen types	type 3 fimbriae		Type 1 fimbriae	High-pathogenicity island: Yersiniabactin			IroA System: Salmochelin synthesis	Fep-Ent System: Enterobactin			
				mrkABCDF	*mrkHIJ*	*fimABCDEFGHIK*	*fyuA*	*irp1 / 2*	*ybtAEPQSTUX*	*iroE*	*fepABCDG*	*fes*	*ybdA*	*entABCEF*
KN1002	11	KL27	O2v2	1	1	1	1	1	1	1	1	1	1	1
KN12786	11	KL107	O3b	1	1	1	0	0	0	1	1	0	1	1
KN13096	258	KL106	O2v2	0	0	1	0	0	0	1	1	1	1	1
KN1328423	258	KL106	O2v2	0	0	1	0	0	0	1	1	1	1	1
KN1400178	258	KL107	O2v2	1	1	1	0	0	0	1	1	1	1	1
KN1400180	258	KL107	O2v2	1	1	1	1	1	1	1	1	1	1	1
KN1400182	258	KL107	O2v1	1	1	1	1	1	1	1	1	1	1	1
KN14299	258	KL107	O2v2	1	0	1	0	0	0	1	1	1	1	1
KN14368	258	KL106	O2v2	0	0	1	0	0	0	1	1	1	1	1
KN1757	258	KL106	O2v2	0	0	0	1	1	1	1	1	1	1	1
KN18517	11	KL13	O3b	1	1	1	0	0	0	1	1	1	1	1
KN18530	11	KL13	O3b	1	1	1	0	0	0	1	1	1	1	1
KN18689	11	KL64	O2v1	1	1	1	1	1	1	1	1	1	1	1
KN18694	11	KL107	O2v1	1	1	1	1	1	1	1	1	1	1	1
KN1950	258	KL107	O2v2	0	0	1	0	0	0	1	1	1	1	1
KN19860	11	KL64	O2v1	1	1	1	1	1	1	1	1	1	1	1
KN19872	11	KL47	OL101	1	1	1	1	1	1	1	1	1	1	1
KN19873	11			1	1	1	1	1	1	1	1	1	1	1
KN19874	11			1	1	1	1	1	1	1	1	1	1	1
KN20456	11			1	1	1	1	1	1	1	1	1	1	1
KN20499	11			1	1	1	0	0	0	1	1	1	1	1
KN20569	11			1	1	1	0	0	0	1	1	1	1	1
KN20662	11			1	1	1	1	1	1	1	1	1	1	1
KN20804	11			1	1	1	1	1	1	1	1	1	1	1
KN21178	11			1	1	1	1	1	1	1	1	1	1	1
KN21217	11			1	1	1	1	1	1	1	1	1	1	1

KN21221	11	1	1	1	0	0	0	1	1	1	1	1
KN21224	11	1	1	1	0	0	0	1	1	1	1	1
KN21306	11	1	1	1	1	1	1	1	1	1	1	1
KN21316	11	1	1	1	1	1	1	1	1	1	1	1
KN21317	11	1	1	1	1	1	1	1	1	1	1	1
KN21320	11	1	1	1	1	1	1	1	1	1	1	1
KN21321	11	1	1	1	1	1	1	1	1	1	1	1
KN21331	11	1	1	1	1	1	1	1	1	1	1	1
KN21333	11	1	1	1	1	1	1	1	1	1	1	1
KN21341	11	1	1	1	1	1	1	1	1	1	1	1
KN21345	11	1	1	1	1	1	1	1	1	1	1	1
KN21352	11	1	1	1	0	0	0	1	1	1	1	1
KN21376	11	1	1	1	0	0	0	1	1	1	1	1
KN21378	11	1	1	1	1	1	1	1	1	1	1	1
KN21384	11	1	1	1	1	1	1	1	1	1	1	1
KN21387	11	1	1	1	0	0	0	1	1	1	1	1
KN21388	11	1	1	1	0	0	0	1	1	1	1	1
KN21391	11	1	1	1	1	1	1	1	1	1	1	1
KN21400	11	1	1	1	0	0	0	1	1	1	1	1
KN21403	11	1	1	1	1	1	1	1	1	1	1	1
KN21411	11	1	1	1	0	0	0	1	1	1	1	1
KN21412	11	1	1	1	0	0	0	1	1	1	1	1
KN21413	11	1	1	1	0	0	0	1	1	1	1	1
KN21420	11	1	1	1	0	0	0	1	1	1	1	1
KN21423	11	1	1	1	0	0	0	1	1	1	1	1
KN21445	11	1	1	1	1	1	1	1	1	1	1	1
KN21447	11	1	1	1	1	1	1	1	1	1	1	1
KN21458	11	1	1	0	0	0	0	1	1	1	1	1
KN21466	11	1	1	1	1	1	1	1	1	1	1	1
KN21507	11	1	1	1	1	1	1	1	1	1	1	1
KN21511	11	1	1	1	1	1	1	1	1	1	1	1
KN21556	11	1	1	1	1	1	1	1	1	1	1	1
KN21570	11	1	1	1	0	0	0	1	1	1	1	1
KN21932	11	1	1	1	0	0	0	1	1	1	1	1
KN21967	11	1	1	0	1	1	1	1	1	1	1	1

KN22002	11	1	0	1	0	0	0	1	1	1	1	1
KN22010	11	1	0	1	0	0	0	1	1	1	1	1
KN22370	11	1	1	1	1	1	1	1	1	1	1	1
KN22384	11	1	1	1	1	1	1	1	1	1	1	1
KN22431	11	1	1	1	1	1	1	1	1	1	1	1
KN22452	11	1	1	1	1		1	1	1	1	1	1
KN22569	11	1	0	1	1	1	1	1	1	1	1	1
KN22570	11	0	0	1	1	1	1	1	1	1	1	1
KN22572	11	1	1	1	1	1	1	1	1	1	1	1
KN2323	11	1	1	1	1	1	1	1	1	1	1	1
KN24396	11	1	1	1	1	1	1	1	1	1	1	1
KN5623	258	1	0	1	1	1	1	1	1	1	1	1
KN5636	11	1	1	1	1	1	1	1	1	1	1	1
KN5647	258	1	0	1	1	1	1	1	1	1	1	1
KN5794	11	1	1	1	1	1	1	1	1	1	1	1
KN7016	11	1	1	1	1	1	1	1	1	1	1	1
KN7018	11	1	1	1	1	1	1	1	1	1	1	1
KN7212	11	1	1	1	1	1	1	1	1	1	1	1
KN7213	11	1	1	1	1	1	1	1	1	1	1	1
KN935	11	1	1	1	0	0	0	1	1	1	1	1
KN942	11	1	1	1	1	1	1	1	1	1	1	1
KN943	11	1	1	1	1	1	1	1	1	1	1	1
KN971	11	1	1	1	1	1	1	1	1	1	1	1
KN976	11	1	0	1	1	1	1	1	1	1	1	1
